# Supplementary material for: Cryo-EM structures of the SARS-CoV-2 endoribonuclease Nsp15 reveal insight into nuclease specificity and dynamics
Source: Nat Commun. 2021 Jan 27;12:636. doi: 10.1038/s41467-020-20608-z (PMC7840905; doi:10.1038/s41467-020-20608-z)
Supplement: Supplementary file 1 — Supplementary Information [file 41467_2020_20608_MOESM1_ESM.pdf]

Supplementary Information

Cryo-EM Structures of the SARS-CoV-2 Endoribonuclease Nsp15 Reveal Insight into Nuclease Specificity and Dynamics

Monica C. Pillon[1,*,†], Meredith N. Frazier[1,†], Lucas B. Dillard[2,†], Jason G. Williams[3], Seda Kocaman[1], Juno M. Krahn[2], Lalith Perera[2], Cassandra K. Hayne[1], Jacob Gordon[1,4,5,6], Zachary D. Stewart[1], Mack Sobhany[1], Leesa J. Deterding[3], Allen L. Hsu[2], Venkata P. Dandey[2], Mario J. Borgnia[2], and Robin E. Stanley[1,*]

[1]Signal Transduction Laboratory, National Institute of Environmental Health Sciences, National Institutes of Health, Department of Health and Human Services, 111 T. W. Alexander Drive, Research Triangle Park, NC 27709, USA

[2]Genome Integrity and Structural Biology Laboratory, National Institute of Environmental Health Sciences, National Institutes of Health, Department of Health and Human Services, 111 T. W. Alexander Drive, Research Triangle Park, NC 27709, USA

[3]Epigenetics and Stem Cell Biology Laboratory, National Institute of Environmental Health Sciences, National Institutes of Health, Department of Health and Human Services, 111 T. W. Alexander Drive, Research Triangle Park, NC 27709, USA

[4]Cambridge Institute for Medical Research, Cambridge, United Kingdom

[5]Department of Haematology, University of Cambridge, Cambridge, United Kingdom

[5]Wellcome Trust-Medical Research Council Stem Cell Institute, University of Cambridge, Cambridge, United Kingdom

[†] These authors contributed equally: Monica C. Pillon, Meredith N. Frazier, Lucas B. Dillard

*Correspondence and requests for materials should be addressed to (email: monica.pillon@nih.gov; robin.stanley@nih.gov)

This PDF file includes:

Supplementary Figures 1 to 12
Supplementary Tables 1 to 4

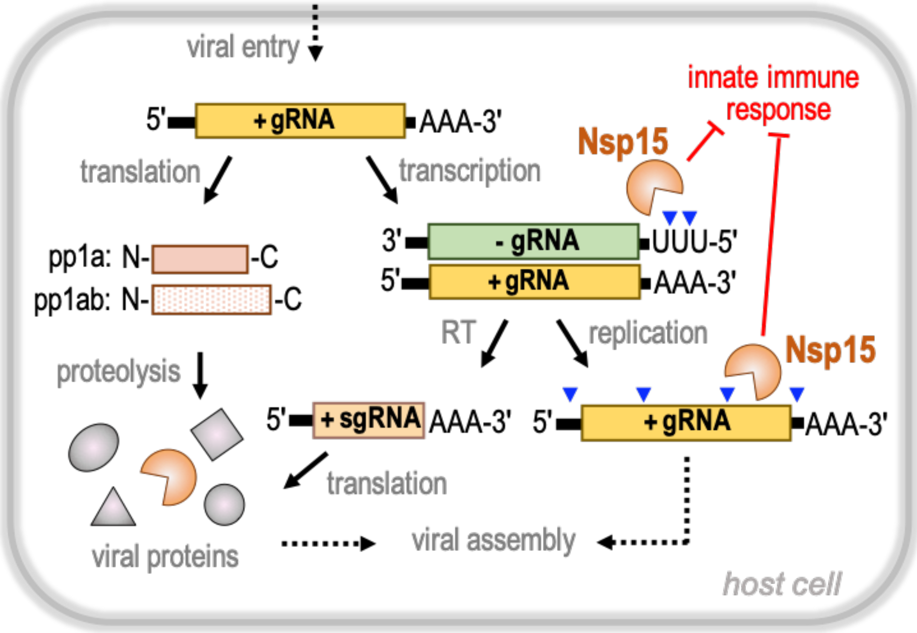

Supplementary Fig. 1. Nsp15 is a central regulator of SARS-CoV-2 RNA processing. Upon viral entry, the single-strand SARS-CoV-2 positive-sense genomic RNA (+gRNA; yellow) is released into the cytoplasm and translated by host ribosomes to generate viral polyproteins pp1a and pp1ab. Subsequent proteolytic cleavage of the polyproteins results in a variety of non-structural proteins (Nsp) essential for diverse viral functions. Transcription of the positive-sense genomic RNA produces a negative-sense genomic RNA (-gRNA; green) intermediate. The negative-sense strand is a template for reverse transcription (RT) generating a series of positive-sense subgenomic RNAs (+sgRNA; brown), which are translated into diverse structural proteins. In addition, the negative-sense strand is also the template for viral replication. The non-structural protein Nsp15 (orange) is a poly-(U) specific endonuclease that cleaves 3′ to uridines within the viral genomic RNA. Nsp15 cleavage sites (blue arrowheads) have been mapped all along the positive-sense genomic RNA and 5′-end of the negative-sense genomic RNA. Nsp15 viral RNA processing plays an important role for evading detection by the host innate immune response.

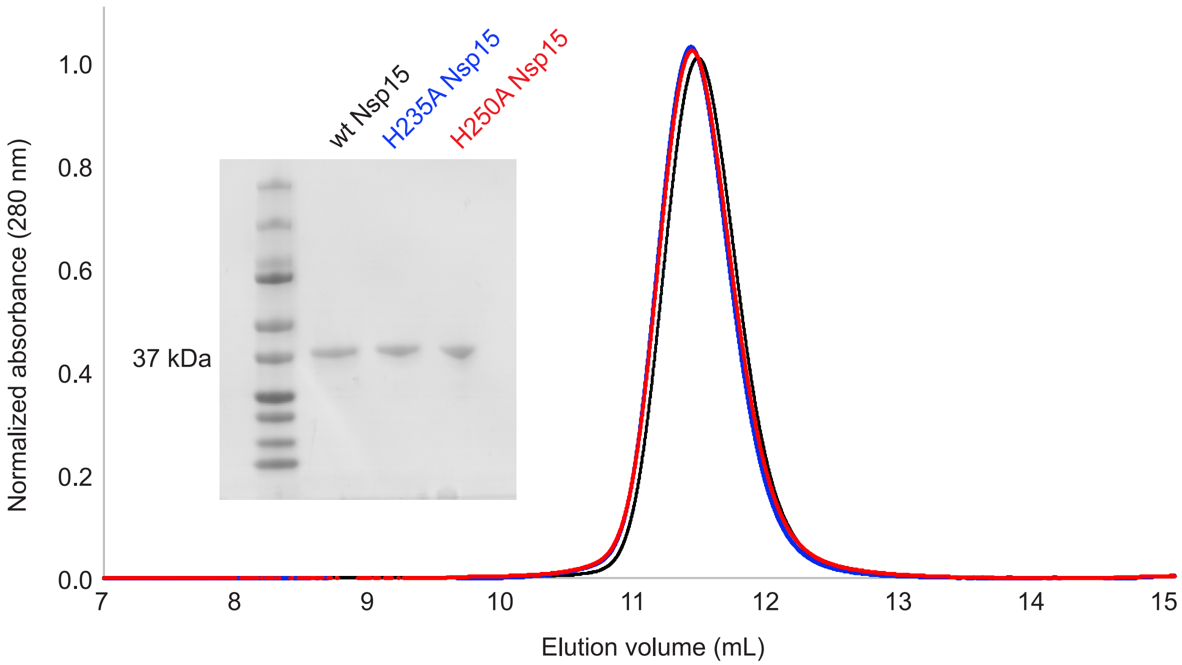

Supplementary Fig. 2. Purification of recombinant SARS-CoV-2 Nsp15 variants. Purified Nsp15 variants wild-type (black), H235A (blue), and H250A (red) were resolved over a Superdex-200 increase 10/300 GL gel filtration column using SEC buffer. Inset is an SDS-PAGE analysis of recombinant Nsp15 wild-type (wt), H235A, and H250A variants. Purifications were performed at least three times for each construct.

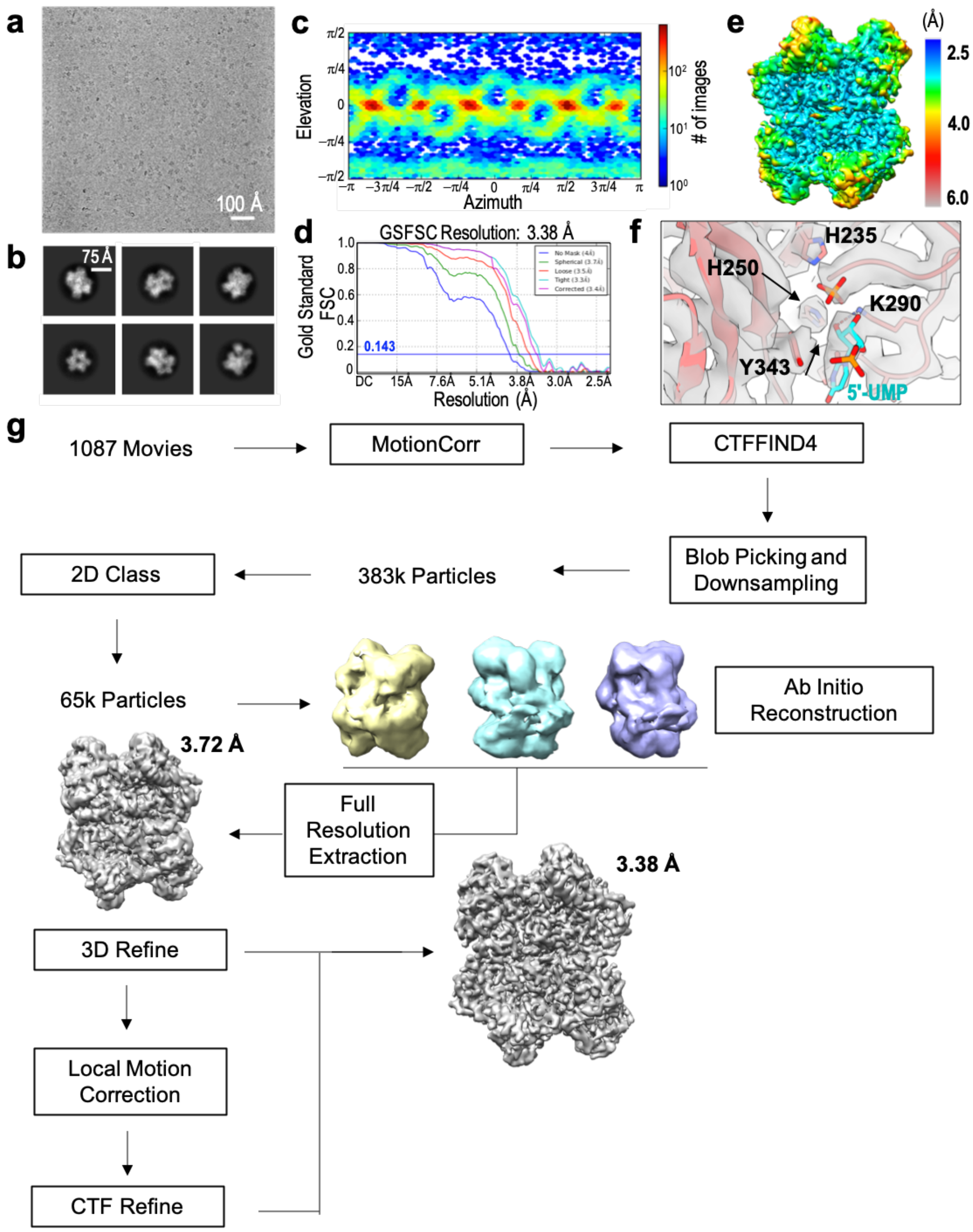

Supplementary Fig. 3. Overview of cryo-EM processing scheme for UTP-bound Nsp15. a A representative micrograph of wt-Nsp15 in the presence of excess UTP in vitreous ice. Four technical replicates of SARS-CoV-2 UTP-bound Nsp15 sample were imaged with similar results. **b** Selected 2D classes generated from 1087 movies collected from an UltrAuFoil R1.2/1.3 300 mesh grid. **c** Angular distribution of UTP-bound wt-Nsp15 particles. **d** Fourier shell correlation (FSC) curve for the UTP-bound Nsp15 reconstruction. The overall resolution is 3.38 Å according to the FSC 0.143 criteria [1,2]. **e** Cryo-EM reconstruction of UTP-bound Nsp15 colored based on local resolution calculated using cryoSparc v2 [3]. **f** Cryo-EM density for Nsp15 endoU active site (gray) with the corresponding model shown as a cartoon with individual active site residues, 5′-UMP, and modeled phosphate shown as sticks. **g** Cryo-EM processing workflow. Picked particles (383,271) were subjected to 2D classification, 3D classification, and refinement prior to local refinement of a single Nsp15 protomer in cryoSPARC v2 [3].

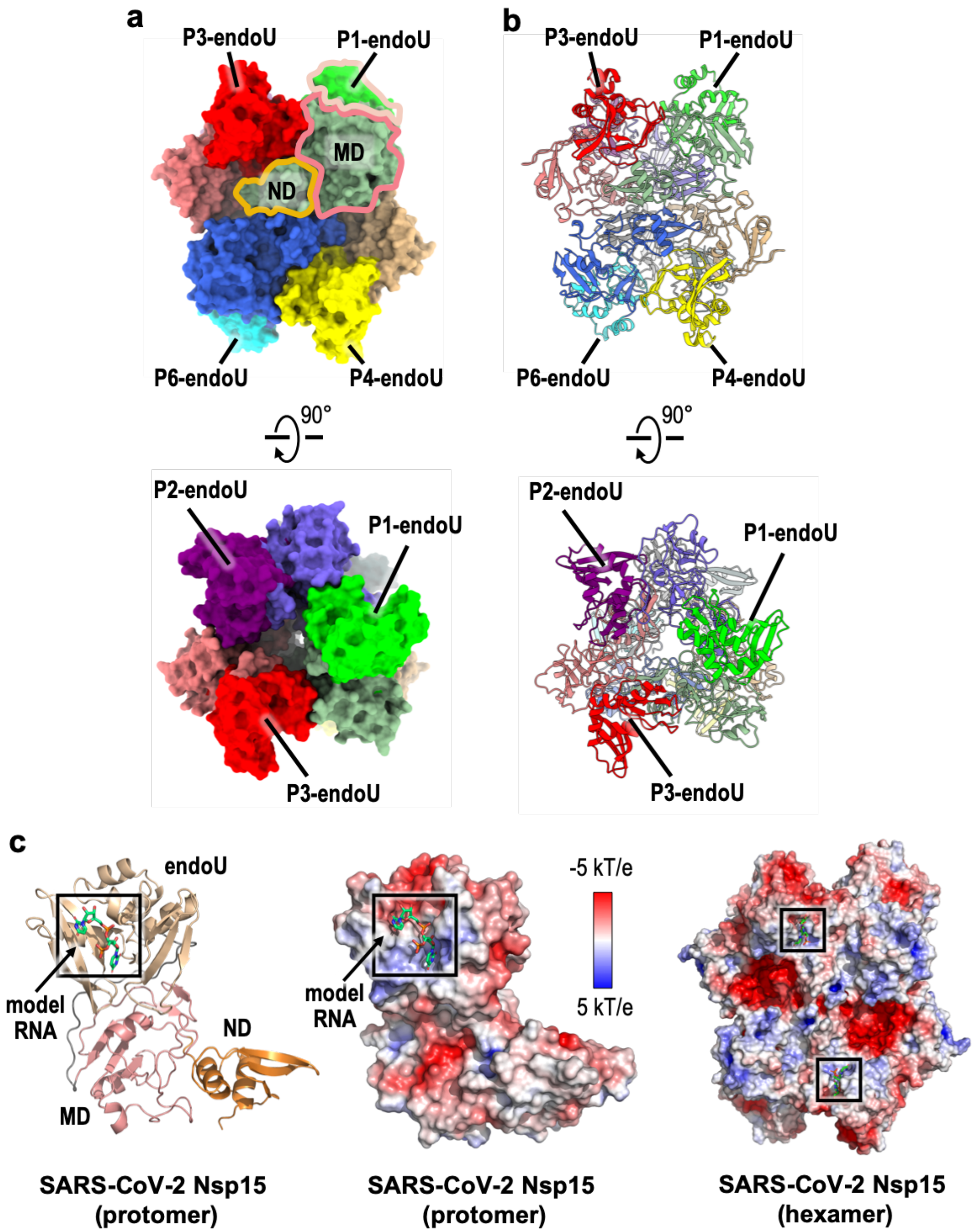

Supplementary Fig. 4. Arrangement of Nsp15 endoU domains. Orthogonal views of **a** surface rendering and **b** cartoon model of hexameric Nsp15. The ND, MD, and endoU domains are outlined in orange, red, and beige, respectfully. The Nsp15 ND and MD domains are colored as seen in Fig. 2B. The endoU domains of six Nsp15 protomers (P1 to P6) are colored in bright hues of green, purple, red, yellow, gray, and cyan, respectfully. **c** Cartoon model (left) and corresponding electrostatic surface potential of an Nsp15 protomer (middle) and hexamer (right). Dinucleotide RNA substrate is modeled into the Nsp15 endoribonuclease active sites and shown as green sticks. The dinucleotide model was created based on the positioning the bridging phosphate interaction with H250 and alignment with the attaching O2′. The 3′ nucleotide was oriented for base stacking with W333.

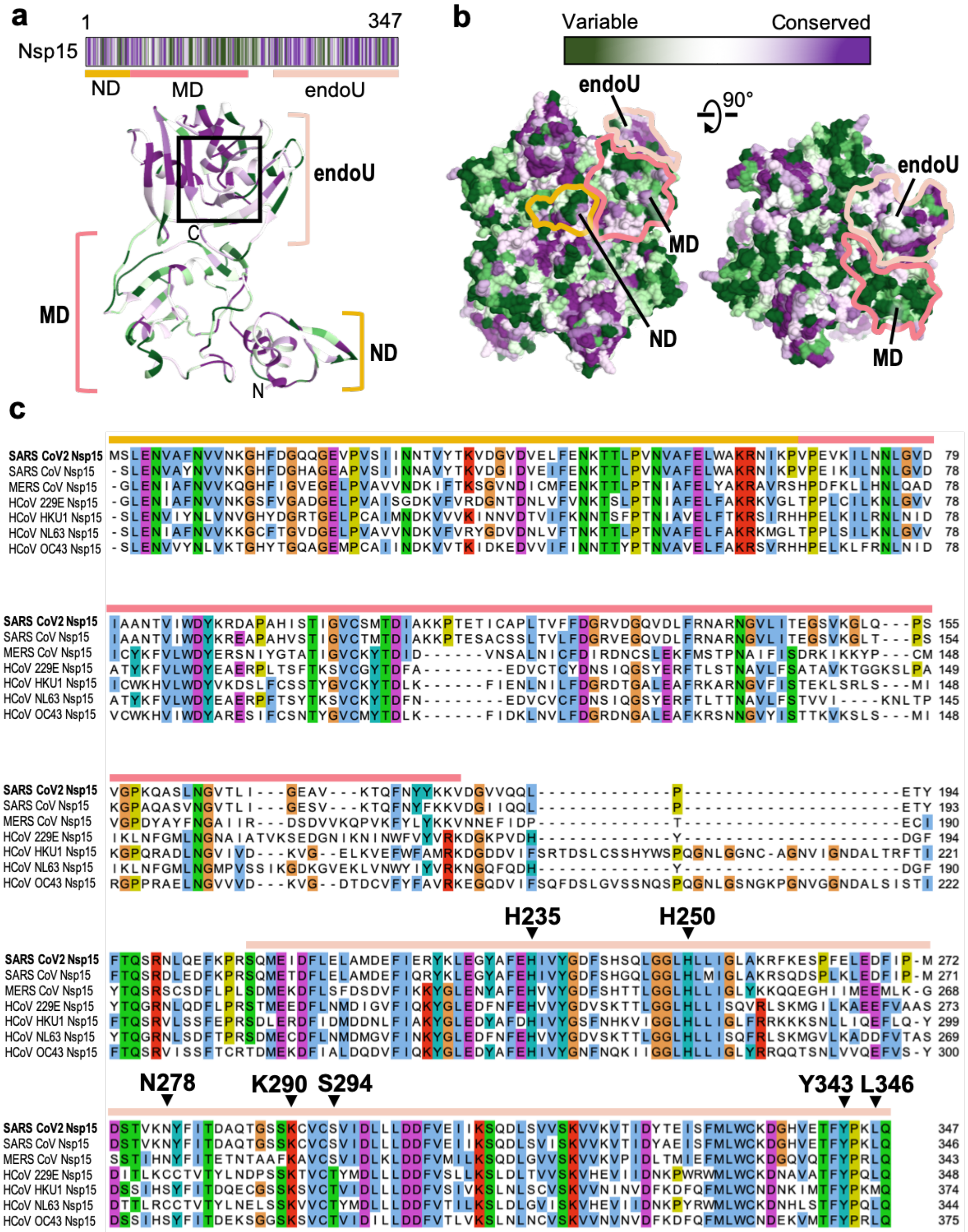

Supplementary Fig. 5. Sequence alignment and conservation of Nsp15. a Consurf analysis (http:// consurf-hssp.tau.ac.il) of Nsp15 homologues plotted with respect to its domain architecture. Conserved and variable amino acid residues are colored as purple and green bars, respectively. The SARS-CoV-2 Nsp15 model of the protomer is shown as a cartoon and colored based on amino acid residue conservation. **b** Surface representation of hexameric Nsp15 illustrating conserved (purple) to variable (green) residues based on the Consurf analysis. The ND, MD, and endoU domains are outlined in orange, red, and beige, respectfully. **c** Sequence alignment of Nsp15 performed by PROMAL3D[4] and illustrated by JalView[5]. Black arrowheads mark the position of Nsp15 endoU residues located within the active site.

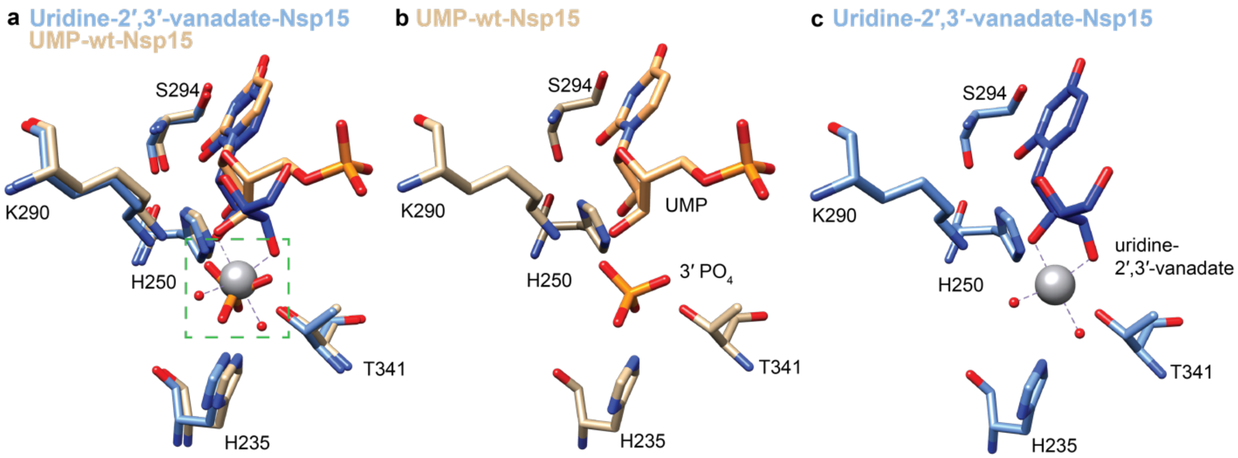

Supplementary Fig. 6. Model phosphate alignment. An unpublished structure (PDB: 7K1L) was deposited in the PDB during revision of this manuscript. This structure represents a mimic of the cyclic phosphate intermediate, and **a** superposition with our structure (PDB: 7K0R) reveals good agreement between the uridine-2′,3′-vanadate and our placement of the model phosphate. **b and c** The standalone structures superposed in **a**.

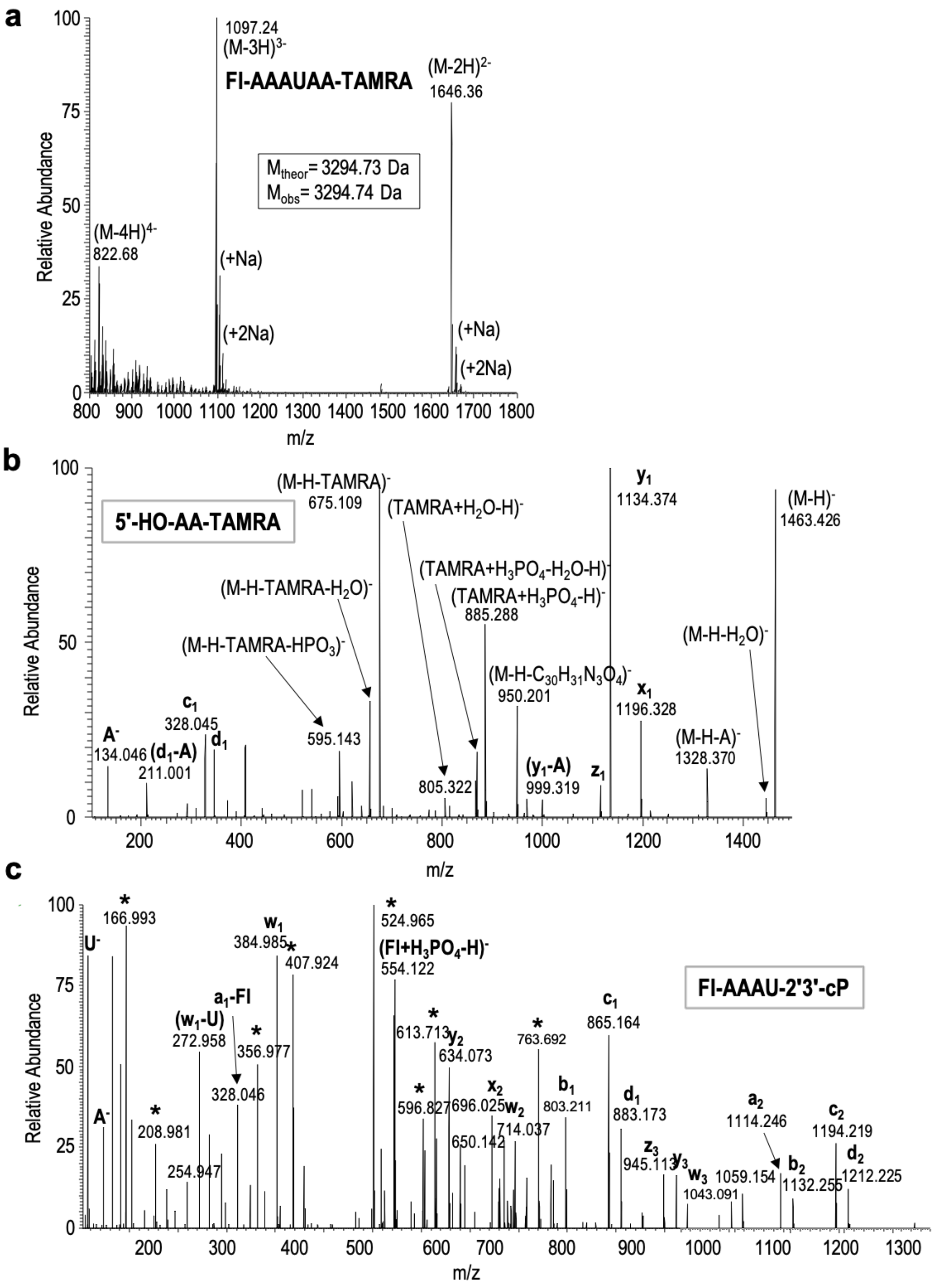

Supplementary Fig. 7. RNA analysis using mass spectrometry. **a** The MS spectrum of the uncut FI-AAAUAA-TAMRA synthetic oligonucleotide shows doubly and triply charged species that correspond in mass to the theoretical mass of the intact oligonucleotide. **b** The MSMS spectrum of the m/z 1463.42 ion yields extensive fragmentation[6] that confirms the identity of 5′-HO-AA-TAMRA cleavage product. **c** The MSMS spectrum of the m/z 914.14 ion yields extensive fragmentation[6] that confirms the identity of FI-AAAU-2′3′-cP cleavage product. This spectrum is chimeric due to a coeluting, co-isolating molecule(s). Peaks labeled with asterisks (*) arise from coeluting, co-isolating species as determined by extracted ion chromatograms generated for the various fragment ions in the 914.14 channel. Fragment ions that have a distinct peak at 6.2 minutes were assumed to arise from the 5′-FI-AAAU-2′3′-cP cleavage product.

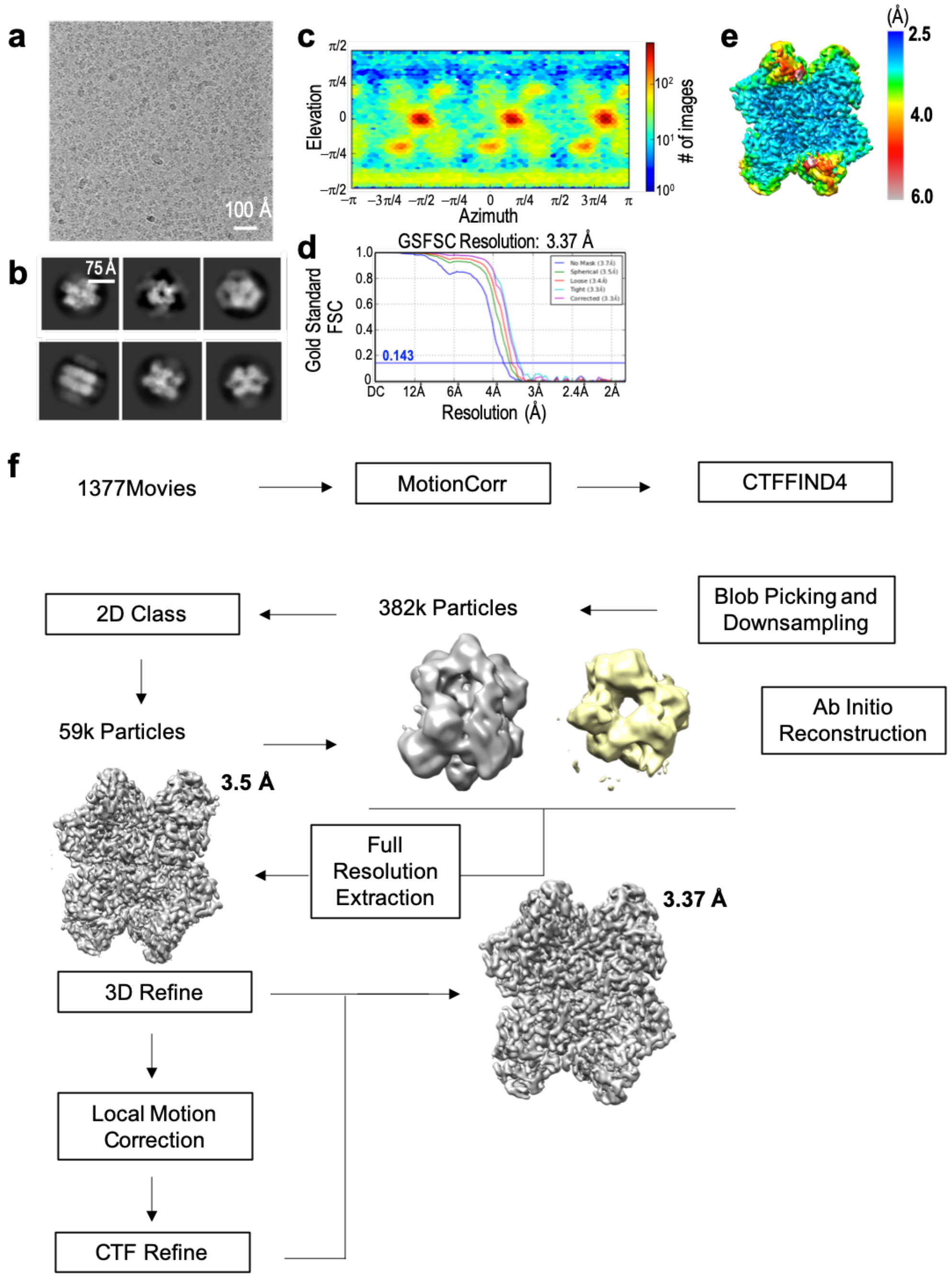

Supplementary Fig. 8. Overview of cryo-EM processing scheme for apo-state Nsp15 H235A dataset i. a A representative micrograph of apo-state Nsp15 H235A in vitreous ice. Four technical replicates of SARS-CoV-2 Nsp15 H235A sample were imaged with similar results. **b** Selected 2D classes generated from 1377 movies collected from an UltrAuFoil R1.2/1.3 300 mesh grid. **c** Angular distribution of apo-state Nsp15 H235A particles. **d** Fourier shell correlation (FSC) curve for the apo-state Nsp15 H235A reconstruction. The overall resolution is 3.30 Å according to the FSC 0.143 criteria[1,2]. **e** Cryo-EM reconstruction of apo-state Nsp15 H235A colored based on local resolution calculated using cryoSparc v2[3]. **f** Cryo-EM processing workflow. Picked particles (382,348) were subjected to 2D classification, 3D classification, and refinement prior to local refinement of a single Nsp15 protomer in cryoSPARC v2 [3].

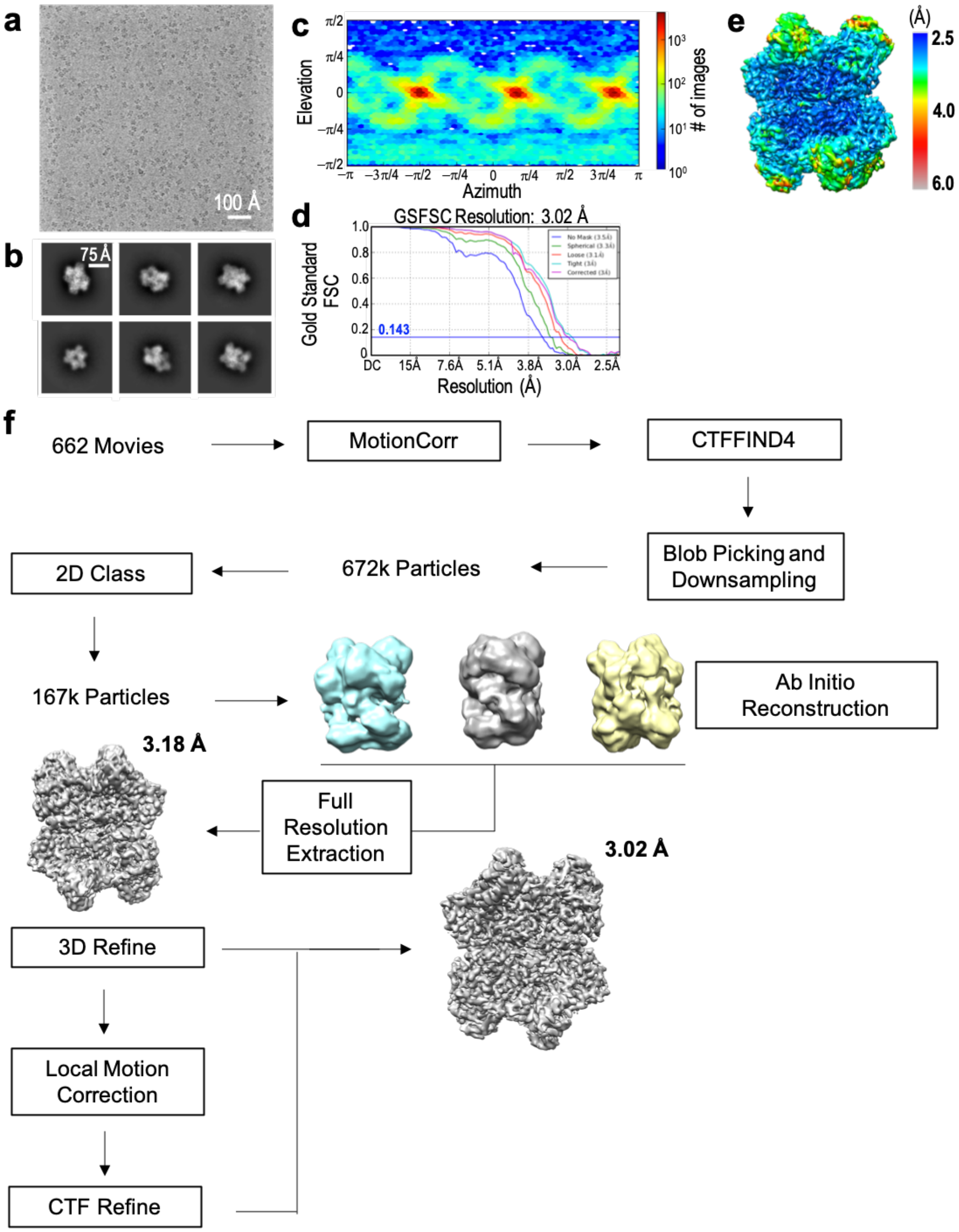

Supplementary Fig. 9. Overview of cryo-EM processing scheme for apo-state wt Nsp15. a A representative micrograph of apo-state wt-Nsp15 in vitreous ice. Four technical replicates of SARS-CoV-2 wt Nsp15 sample were imaged with similar results. **b** Selected 2D classes generated from 662 movies collected from an UltrAuFoil R1.2/1.3 300 mesh grid. **c** Angular distribution of apo-state wt-Nsp15 particles. **d** Fourier shell correlation (FSC) curve for the apo-state Nsp15 reconstruction. The overall resolution is 3.02 Å according to the FSC 0.143 criteria[1,2]. **e** Cryo-EM reconstruction of apo-state Nsp15 colored based on local resolution calculated using cryoSparc v2[3]. **f** Cryo-EM processing workflow. Picked particles (672,879) were subjected to 2D classification, 3D classification, and refinement prior to local refinement of a single Nsp15 protomer in cryoSPARC v2 [3].

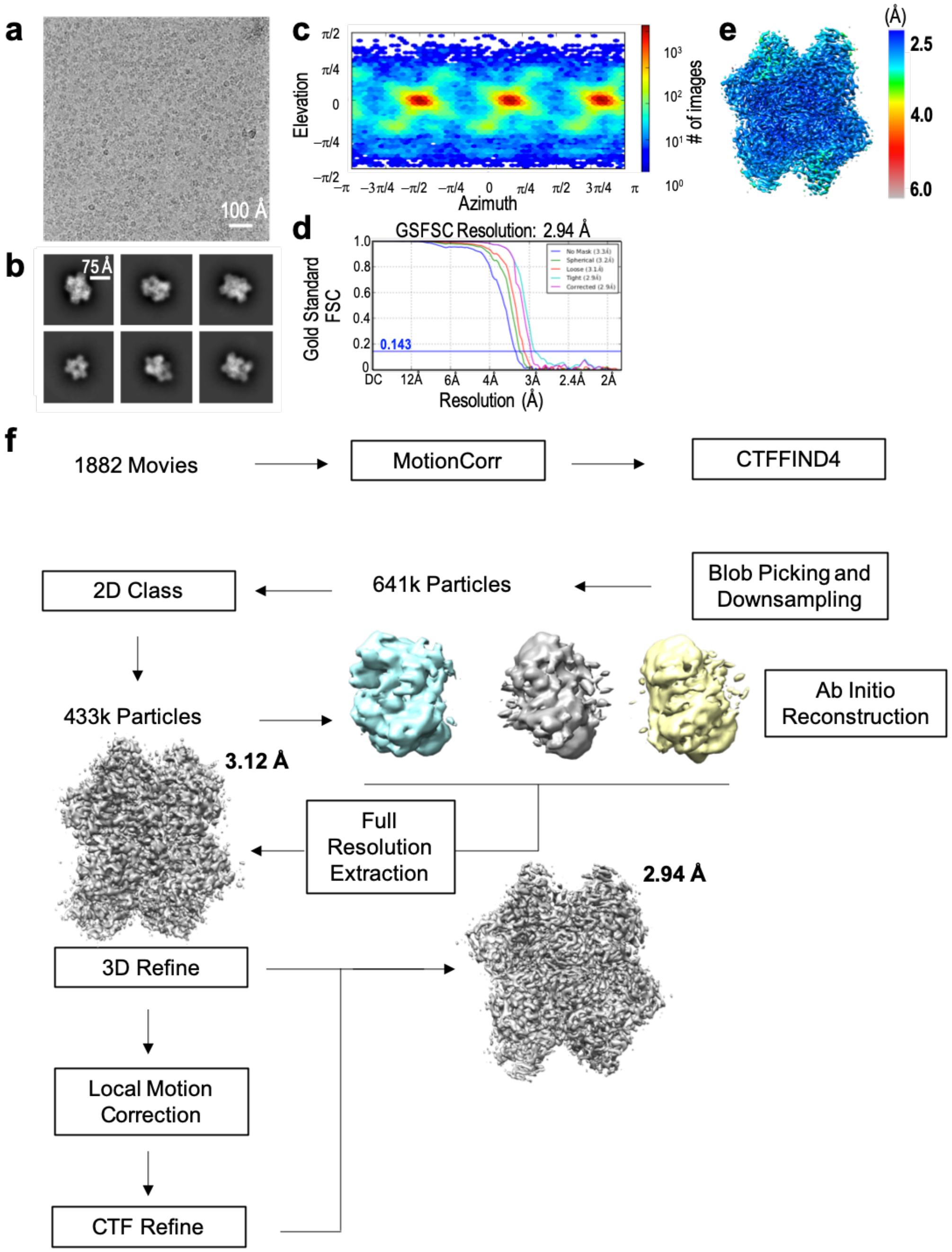

Supplementary Fig. 10. Overview of cryo-EM processing scheme for apo-state Nsp15 H235A dataset ii. a A representative micrograph of apo-state Nsp15 H235A in vitreous ice. Four technical replicates of SARS-CoV-2 Nsp15 H235A sample were imaged with similar results. **b** Selected 2D classes generated from 1882 movies collected from an UltrAuFoil R1.2/1.3 300 mesh grid. **c** Angular distribution of apo-state Nsp15 H235A particles. **d** Fourier shell correlation (FSC) curve for the apo-state Nsp15 reconstruction. The overall resolution is 2.94 Å according to the FSC 0.143 criteria[1,2]. **e** Cryo-EM reconstruction of apo-state Nsp15 H235A colored based on local resolution calculated using cryoSparc[3]. **f** Cryo-EM processing workflow. Picked particles (641,748) were subjected to 2D classification, 3D classification, and refinement prior to local refinement of a single Nsp15 protomer in cryoSPARC v2[3].

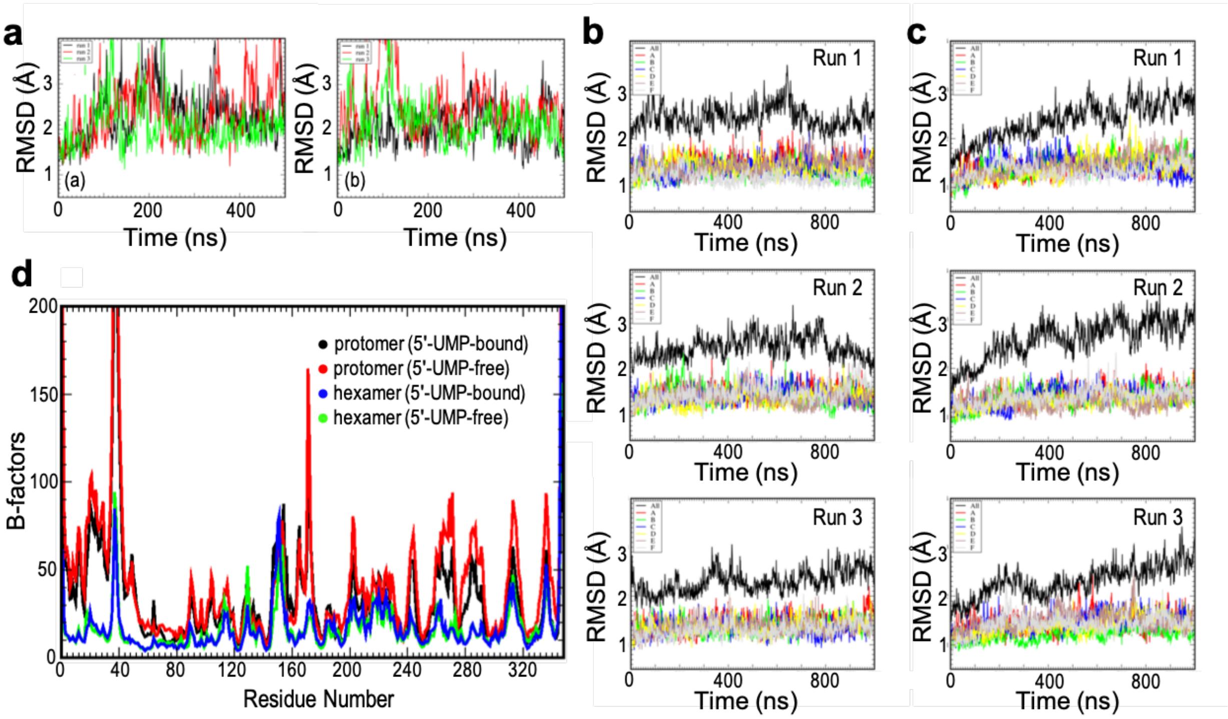

Supplementary Fig. 11. Molecular dynamics of Nsp15. a Root mean square deviations (RMSDs) from three runs (black, red, and green) of a substrate-free monomer (left) and 5′-UMP-bound monomer (right). Root mean square deviations (RMSDs) from three replicates of **b** substrate-free protomers from the hexamer (black= hexamer RMSD; colored plots= individual monomer RMSDs) and **c** 5′-UMP-bound protomers from the hexamer (black= hexamer RMSD; colored plots= individual monomer RMSDs). Individual protomer RMSDs from hexamer systems are smaller when compared with the values of monomer systems, due to subdued dynamics of monomer in the hexameric configurations. **d** B-factors calculated from the positional fluctuations of the backbone atoms (red= substrate-free monomer; black= 5′-UMP-bound monomer; green= a substrate-free monomer from the hexamer; blue= a 5′-UMP-bound monomer from the hexamer).

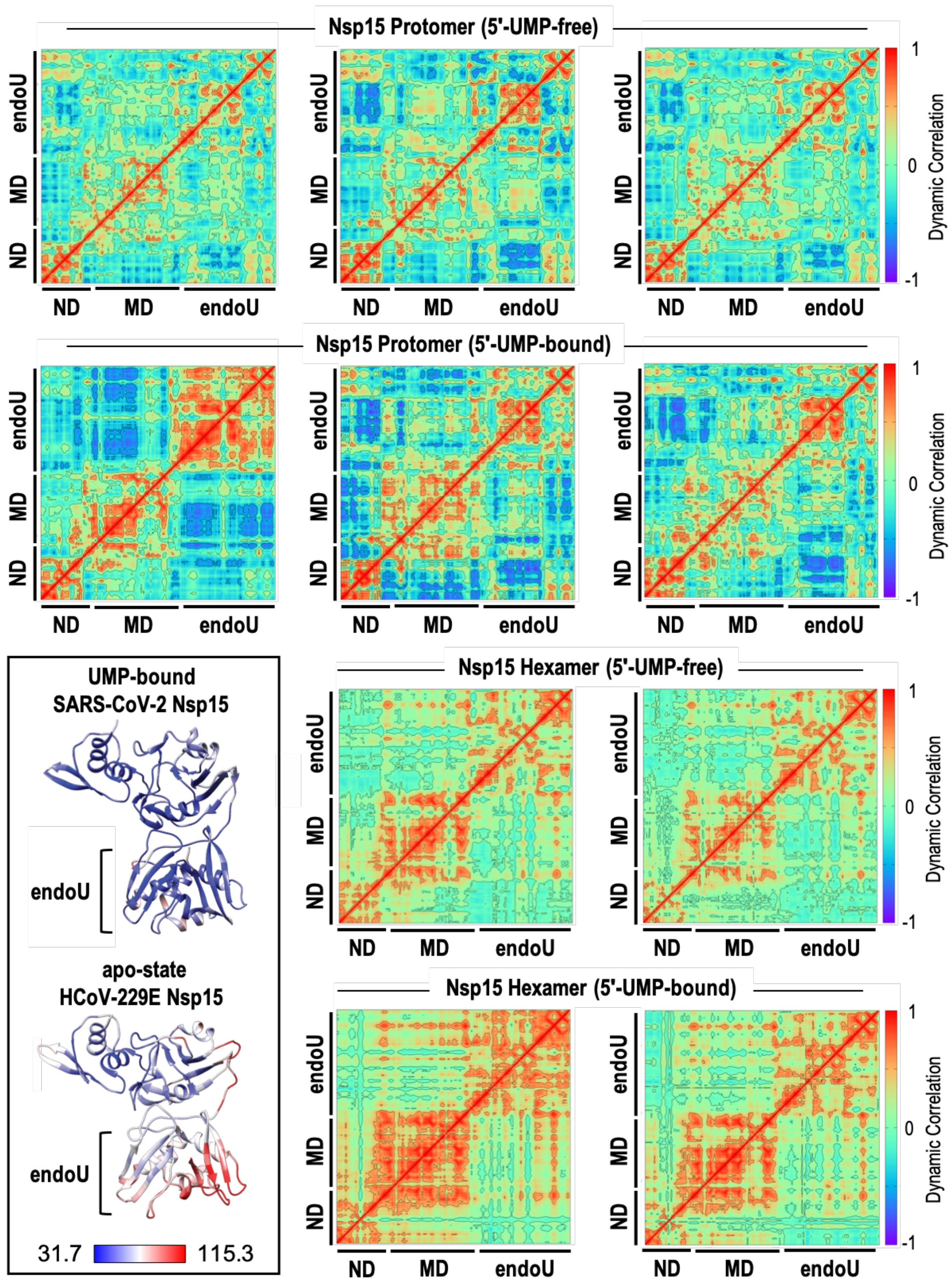

Supplementary Fig. 12. Nsp15 Molecular Dynamics Simulations. Dynamical cross-correlation matrices (DCCM) from 2-3 independent runs for a 5′-UMP-free Nsp15 monomer, a 5′-UMP-bound Nsp15 monomer, a 5′-UMP-free monomer from the Nsp15 hexamer, and a 5′-UMP-bound monomer from the Nsp15 hexamer. Inset features the models of UMP-bound SARS-CoV-2 Nsp15 (PDB ID 6WLC) and apo-state HCoV-229E Nsp15 (PDB ID 4RS4) determined using crystallography. Ribbon diagram is colored according to average B factor value where blue is 31.7, white is 73.5, and red is 115.3.

Supplementary Table 1. Theoretical masses of putative Nsp15 RNA cleavage products.

	Mass (Da)	m/z (M-H)$^-$	m/z (M-2H)$^{2-}$	m/z (M-3H)$^{3-}$
Fl-A-3'OH	804.215	803.207	401.100	267.064
Fl-AA-3'OH	1133.267	1132.259	565.626	376.748
Fl-AAA-3'OH	1462.319	1461.311	730.152	486.432
Fl-AAAU-3'OH	1768.344	1767.336	883.164	588.440
Fl-AAAUA-3'OH	2097.397	2096.389	1047.691	698.125
Fl-AAAUAA-3'OH	2426.449	2425.441	1212.217	807.809
Fl-A-3'P	884.181	883.173	441.083	293.719
Fl-AA-3'P	1213.233	1212.225	605.609	403.403
Fl-AAA-3'P	1542.285	1541.277	770.135	513.087
Fl-AAAU-3'P	1848.310	1847.302	923.147	615.096
Fl-AAAUA-3'P	2177.363	2176.355	1087.674	724.780
Fl-AAAUAA-3'P	2506.415	2505.407	1252.200	834.464
Fl-A-2'3'-cP	866.171	865.163	432.078	287.716
Fl-AA-2'3'-cP	1195.223	1194.215	596.604	397.400
Fl-AAA-2'3'-cP	1524.275	1523.267	761.130	507.084
Fl-AAAU-2'3'-cP	1830.300	1829.292	914.142	609.092
Fl-AAAUA-2'3'-cP	2159.353	2158.345	1078.669	718.777
Fl-AAAUAA-2'3'-cP	2488.405	2487.397	1243.195	828.461
Fl-AAAUAA-TAMRA	3294.734	3293.726	1646.359	1097.237
5'HO-AAAUAA-TAMRA	2757.615	2756.607	1377.800	918.197
5'HO-AAUAA-TAMRA	2428.563	2427.555	1213.274	808.513
5'HO-AUAA-TAMRA	2099.510	2098.502	1048.747	698.829
5'HO-UAA-TAMRA	1770.458	1769.450	884.221	589.145
5'HO-AA-TAMRA	1464.433	1463.425	731.209	487.137
5'HO-A-TAMRA	1135.381	1134.373	566.683	377.453
5'P-AAAUAA-TAMRA	2837.581	2836.573	1417.783	944.853
5'P-AAUAA-TAMRA	2508.529	2507.521	1253.257	835.169
5'P-AUAA-TAMRA	2179.476	2178.468	1088.730	725.484
5'P-UAA-TAMRA	1850.424	1849.416	924.204	615.800
5'P-AA-TAMRA	1544.399	1543.391	771.192	513.792
5'P-A-TAMRA	1215.347	1214.339	606.666	404.108

Supplementary Table 2. Average distance between SARS-CoV-2 Nsp15 active site residues.

Nsp15 Model*		Average distance between select atoms, Å (standard deviation): H235-CB	H250-CB	K290-NZ	V292-O	S294-OG	Y343-CG	L346-N
xray	**H235-CB**	0.000(0.000)	9.158(0.000)	8.673(0.000)	13.400(0.000)	14.769(0.000)	10.066(0.000)	17.471(0.000)
Protomer 5'-UMP-free	**H235-CB**	0.000(0.000)	9.490(0.858)	10.752(1.353)	15.010(1.076)	14.971(1.346)	10.694(0.987)	17.949(1.062)
Protomer 5'-UMP-bound	**H235-CB**	0.000(0.000)	9.398(0.341)	9.394(1.198)	13.777(0.775)	15.122(0.886)	11.193(0.621)	18.354(0.668)
Hexamer-5'-UMP-free	**H235-CB**	0.000(0.000)	9.579(0.945)	10.927(1.285)	15.077(1.028)	15.449(1.239)	10.787(0.922)	17.973(1.086)
Hexamer-5'-UMP-bound	**H235-CB**	0.000(0.000)	9.631(0.662)	9.093(0.984)	13.634(0.859)	15.588(0.826)	11.131(0.779)	18.498(0.825)
xray	**H250-CB**	9.158(0.000)	0.000(0.000)	7.036(0.000)	8.772(0.000)	7.315(0.000)	5.771(0.000)	10.081(0.000)
Protomer 5'-UMP-free	**H250-CB**	9.490(0.858)	0.000(0.000)	8.242(1.480)	9.696(0.658)	6.706(0.966)	6.258(0.688)	10.573(0.752)
Protomer 5'-UMP-bound	**H250-CB**	9.398(0.341)	0.000(0.000)	7.548(1.558)	9.108(0.550)	7.095(0.815)	6.480(0.568)	10.958(0.611)
Hexamer-5'-UMP-free	**H250-CB**	9.579(0.945)	0.000(0.000)	8.493(1.380)	10.015(0.687)	6.960(0.944)	6.502(0.792)	10.645(0.952)
Hexamer-5'-UMP-bound	**H250-CB**	9.631(0.662)	0.000(0.000)	6.842(0.893)	8.896(0.407)	7.436(0.582)	6.267(0.553)	10.700(0.608)
xray	**K290-NZ**	8.673(0.000)	7.036(0.000)	0.000(0.000)	5.098(0.000)	8.357(0.000)	7.207(0.000)	11.633(0.000)
Protomer 5'-UMP-free	**K290-NZ**	10.752(1.353)	8.242(1.480)	0.000(0.000)	5.096(1.049)	8.396(1.512)	7.832(0.977)	11.266(1.352)
Protomer 5'-UMP-bound	**K290-NZ**	9.394(1.198)	7.548(1.558)	0.000(0.000)	4.993(0.851)	8.665(1.177)	7.574(0.802)	11.931(0.913)
Hexamer-5'-UMP-free	**K290-NZ**	10.927(1.285)	8.493(1.380)	0.000(0.000)	4.875(0.933)	8.875(1.392)	7.873(0.936)	11.263(1.442)
Hexamer-5'-UMP-bound	**K290-NZ**	9.093(0.984)	6.842(0.893)	0.000(0.000)	4.776(0.542)	8.510(0.670)	7.236(0.526)	11.890(0.831)
xray	**V292-O**	13.400(0.000)	8.772(0.000)	5.098(0.000)	0.000(0.000)	5.164(0.000)	7.619(0.000)	8.053(0.000)
Protomer 5'-UMP-free	**V292-O**	15.010(1.076)	9.696(0.658)	5.096(1.049)	0.000(0.000)	6.430(1.323)	9.400(0.865)	9.073(1.263)
Protomer 5'-UMP-bound	**V292-O**	13.777(0.775)	9.108(0.550)	4.993(0.851)	0.000(0.000)	6.137(1.062)	8.337(0.849)	9.197(1.054)
Hexamer-5'-UMP-free	**V292-O**	15.077(1.028)	10.015(0.687)	4.875(0.933)	0.000(0.000)	7.143(1.157)	9.757(0.829)	9.657(1.386)
Hexamer-5'-UMP-bound	**V292-O**	13.634(0.859)	8.896(0.407)	4.776(0.542)	0.000(0.000)	6.110(0.598)	8.189(0.605)	9.320(0.890)
xray	**S294-OG**	14.769(0.000)	7.315(0.000)	8.357(0.000)	5.164(0.000)	0.000(0.000)	6.359(0.000)	3.654(0.000)
Protomer 5'-UMP-free	**S294-OG**	14.971(1.346)	6.706(0.966)	8.396(1.512)	6.430(1.323)	0.000(0.000)	6.825(1.224)	5.238(1.095)
Protomer 5'-UMP-bound	**S294-OG**	15.122(0.886)	7.095(0.815)	8.665(1.177)	6.137(1.062)	0.000(0.000)	6.644(0.907)	5.093(1.080)
Hexamer-5'-UMP-free	**S294-OG**	15.449(1.239)	6.960(0.944)	8.875(1.392)	7.143(1.157)	0.000(0.000)	7.473(1.188)	5.498(1.276)
Hexamer-5'-UMP-bound	**S294-OG**	15.588(0.826)	7.436(0.582)	8.510(0.670)	6.110(0.598)	0.000(0.000)	6.768(0.625)	4.422(1.066)
xray	**Y343-CG**	10.066(0.000)	5.771(0.000)	7.207(0.000)	7.619(0.000)	6.359(0.000)	0.000(0.000)	7.871(0.000)
Protomer 5'-UMP-free	**Y343-CG**	10.694(0.987)	6.258(0.688)	7.832(0.977)	9.400(0.865)	6.825(1.224)	0.000(0.000)	7.768(0.705)
Protomer 5'-UMP-bound	**Y343-CG**	11.193(0.621)	6.480(0.568)	7.574(0.802)	8.337(0.849)	6.644(0.907)	0.000(0.000)	7.705(0.481)
Hexamer-5'-UMP-free	**Y343-CG**	10.787(0.922)	6.502(0.792)	7.873(0.936)	9.757(0.829)	7.473(1.188)	0.000(0.000)	7.729(0.688)
Hexamer-5'-UMP-bound	**Y343-CG**	11.131(0.779)	6.267(0.553)	7.236(0.526)	8.189(0.605)	6.768(0.625)	0.000(0.000)	7.945(0.547)
xray	**L346-N**	17.471(0.000)	10.081(0.000)	11.633(0.000)	8.053(0.000)	3.654(0.000)	7.871(0.000)	0.000(0.000)
Protomer 5'-UMP-free	**L346-N**	17.949(1.062)	10.573(0.752)	11.266(1.352)	9.073(1.263)	5.238(1.095)	7.768(0.705)	0.000(0.000)
Protomer 5'-UMP-bound	**L346-N**	18.354(0.668)	10.958(0.611)	11.931(0.913)	9.197(1.054)	5.093(1.080)	7.705(0.481)	0.000(0.000)
Hexamer-5'-UMP-free	**L346-N**	17.973(1.086)	10.645(0.952)	11.263(1.442)	9.657(1.386)	5.498(1.276)	7.729(0.688)	0.000(0.000)
Hexamer-5'-UMP-bound	**L346-N**	18.498(0.825)	10.700(0.608)	11.890(0.831)	9.320(0.890)	4.422(1.066)	7.945(0.547)	0.000(0.000)

*Nsp15 models were the 5'-UMP-bound Nsp15 crystal structure (xray) or the average model from three independent molecular dynamics simulations. PDB ID 6WLC was the reference coordinates used to generate the Nsp15 models.

Supplementary Table 3. Binding free energies estimated using MMGBSA calculations for the 50-ns segments of the final 450 ns of the 5′-UMP-bound Nsp15 hexamer simulation.

Time (ns)	Nsp15 protomers of the hexameric assembly					
	A	B	C	D	E	F
**385-435**	-10.5 ± 3.7	-23.9 ±1.9	-18.6 ± 1.0	-19.1 ± 1.0	-17.7 ± 0.4	-18.7 ± 1.0
**435-485**	4.7 ± 1.9	-15.4 ± 0.9	-18.3 ± 1.0	-15.3 ± 1.4	-19.1 ± 1.1	-14.7 ± 1.1
**485-535**	0.0	-5.4 ± 3.2	-14.6 ± 1.4	-21.3 ± 1.3	-16.7 ± 1.1	-18.4 ± 2.0
**535-585**	0.0	-5.7 ± 2.8	-1.6 ± 1,2	-15.9 ± 1.5	-14.8 ± 1.2	-6.8 ± 3.2
**585-635**	0.0	-8.7 ±3.0	23.2 ± 1.8	-15.6 ± 1.4	-13.2 ± 1.6	-1.4 ± 2.5
**635-685**	0.0	-7.2 ± 2.0	29.4 ± 1.4	-14.2 ± 1.7	-14.7 ± 1.4	4.4 ± 2.0
**685-735**	0.0	-4.3 ± 3.2	34.6 ± 2.0	-16.9 ± 1.0	-8.4 ± 2.5	1.2 ± 3.1
**735-783**	0.0	-1.2 ± 2.2	32.8 ± 2.6	-17.0 ±1.3	-6.2 ± 1.7	-3.3 ± 2.2
**785-835**	0.0	-0.9 ± 2.7	36.1 ± 2.7	-16.7 ± 0.9	-8.2 ± 2.8	-1.8 ± 1.3

Supplementary Table 4. Sequences of codon optimized Nsp15 constructs used in this study.

Construct	Codon optimized sequence
wt-Nsp15	ATGGGCAGCAGCCATCATCATCATCATCACAGCAGCGGCCTGGTGCC<br>GCGCGGCAGCCATATGCTCGAGGAAAACCTGTATTTTCAGTCCCTGGA<br>GATGAGCCTGGAGAACGTTGCGTTTAACGTGGTTAACAAGGGTCACTT<br>CGACGGTCAGCAAGGCGAAGTGCCGGTTAGCATCATTAACAACACCGT<br>GTACACCAAGGTTGACGGCGTGGATGTTGAGCTGTTTGAAAACAAAAC<br>CACCCTGCCGGTGAACGTTGCGTTCGAGCTGTGGGCGAAGCGTAACA<br>TCAAACCGGTGCCGGAAGTTAAGATTCTGAACAACCTGGGTGTGGACA<br>TCGCGGCGAACACCGTTATTTGGGACTATAAACGTGATGCGCCGGCG<br>CACATCAGCACCATTGGCGTTTGCAGCATGACCGACATCGCGAAGAAA<br>CCGACCGAAACCATTTGCGCGCCGCTGACCGTGTTCTTTGACGGTCGT<br>GTGGATGGCCAGGTTGACCTGTTTCGTAACGCGCGTAACGGTGTGCT<br>GATCACCGAGGGTAGCGTTAAAGGCCTGCAGCCGAGCGTGGGTCCGA<br>AACAAGCGAGCCTGAACGGTGTTACCCTGATTGGCGAAGCGGTGAAG<br>ACCCAGTTCAACTACTATAAGAAAGTTGACGGTGTGGTTCAGCAACTG<br>CCGGAAACCTACTTTACCCAGAGCCGTAACCTGCAAGAGTTCAAGCCG<br>CGTAGCCAAATGGAGATCGATTTTCTGGAACTGGCGATGGACGAGTTC<br>ATTGAACGTTACAAACTGGAGGGTTATGCGTTTGAACACATCGTTTACG<br>GCGATTTCAGCCATAGCCAGCTGGGTGGCCTGCACCTGCTGATTGGTC<br>TGGCGAAGCGTTTCAAAGAGAGCCCGTTTGAGCTGGAAGATTTCATCC<br>CGATGGACAGCACCGTGAAGAACTATTTTATTACCGATGCGCAGACCG<br>GCAGCAGCAAATGCGTGTGCAGCGTTATCGACCTGCTGCTGGACGATT<br>TCGTTGAAATCATTAAAAGCCAAGATCTGAGCGTGGTTAGCAAGGTGG<br>TTAAAGTGACCATCGATTACACCGAGATTAGCTTTATGCTGTGGTGCAA<br>GGACGGTCACGTGGAAACCTTCTATCCGAAACTGCAATAA
Nsp15 H235A	ATGGGCAGCAGCCATCATCATCATCATCACAGCAGCGGCCTGGTGCC<br>GCGCGGCAGCCATATGCTCGAGGAAAACCTGTATTTTCAGTCCCTGGA<br>GATGAGCCTGGAGAACGTTGCGTTTAACGTGGTTAACAAGGGTCACTT<br>CGACGGTCAGCAAGGCGAAGTGCCGGTTAGCATCATTAACAACACCGT<br>GTACACCAAGGTTGACGGCGTGGATGTTGAGCTGTTTGAAAACAAAAC<br>CACCCTGCCGGTGAACGTTGCGTTCGAGCTGTGGGCGAAGCGTAACA<br>TCAAACCGGTGCCGGAAGTTAAGATTCTGAACAACCTGGGTGTGGACA<br>TCGCGGCGAACACCGTTATTTGGGACTATAAACGTGATGCGCCGGCG<br>CACATCAGCACCATTGGCGTTTGCAGCATGACCGACATCGCGAAGAAA<br>CCGACCGAAACCATTTGCGCGCCGCTGACCGTGTTCTTTGACGGTCGT<br>GTGGATGGCCAGGTTGACCTGTTTCGTAACGCGCGTAACGGTGTGCT<br>GATCACCGAGGGTAGCGTTAAAGGCCTGCAGCCGAGCGTGGGTCCGA<br>AACAAGCGAGCCTGAACGGTGTTACCCTGATTGGCGAAGCGGTGAAG<br>ACCCAGTTCAACTACTATAAGAAAGTTGACGGTGTGGTTCAGCAACTG<br>CCGGAAACCTACTTTACCCAGAGCCGTAACCTGCAAGAGTTCAAGCCG<br>CGTAGCCAAATGGAGATCGATTTTCTGGAACTGGCGATGGACGAGTTC<br>ATTGAACGTTACAAACTGGAGGGTTATGCGTTTGAAGCGATCGTTTACG<br>GCGATTTCAGCCATAGCCAGCTGGGTGGCCTGCACCTGCTGATTGGTC<br>TGGCGAAGCGTTTCAAAGAGAGCCCGTTTGAGCTGGAAGATTTCATCC<br>CGATGGACAGCACCGTGAAGAACTATTTTATTACCGATGCGCAGACCG<br>GCAGCAGCAAATGCGTGTGCAGCGTTATCGACCTGCTGCTGGACGATT<br>TCGTTGAAATCATTAAAAGCCAAGATCTGAGCGTGGTTAGCAAGGTGG<br>TTAAAGTGACCATCGATTACACCGAGATTAGCTTTATGCTGTGGTGCAA<br>GGACGGTCACGTGGAAACCTTCTATCCGAAACTGCAATAA

Nsp15 H250A	ATGGGCAGCAGCCATCATCATCATCATCACAGCAGCGGCCTGGTGCC<br>GCGCGGCAGCCATATGCTCGAGGAAAACCTGTATTTTCAGTCCCTGGA<br>GATGAGCCTGGAGAACGTTGCGTTTAACGTGGTTAACAAGGGTCACTT<br>CGACGGTCAGCAAGGCGAAGTGCCGGTTAGCATCATTAACAACACCGT<br>GTACACCAAGGTTGACGGCGTGGATGTTGAGCTGTTTGAAAACAAAAC<br>CACCCTGCCGGTGAACGTTGCGTTCGAGCTGTGGGCGAAGCGTAACA<br>TCAAACCGGTGCCGGAAGTTAAGATTCTGAACAACCTGGGTGTGGACA<br>TCGCGGCGAACACCGTTATTTGGGACTATAAACGTGATGCGCCGGCG<br>CACATCAGCACCATTGGCGTTTGCAGCATGACCGACATCGCGAAGAAA<br>CCGACCGAAACCATTTGCGCGCCGCTGACCGTGTTCTTTGACGGTCGT<br>GTGGATGGCCAGGTTGACCTGTTTCGTAACGCGCGTAACGGTGTGCT<br>GATCACCGAGGGTAGCGTTAAAGGCCTGCAGCCGAGCGTGGGTCCGA<br>AACAAGCGAGCCTGAACGGTGTTACCCTGATTGGCGAAGCGGTGAAG<br>ACCCAGTTCAACTACTATAAGAAAGTTGACGGTGTGGTTCAGCAACTG<br>CCGGAAACCTACTTTACCCAGAGCCGTAACCTGCAAGAGTTCAAGCCG<br>CGTAGCCAAATGGAGATCGATTTTCTGGAACTGGCGATGGACGAGTTC<br>ATTGAACGTTACAAACTGGAGGGTTATGCGTTTGAACACATCGTTTACG<br>GCGATTTCAGCCATAGCCAGCTGGGTGGCCTGGCGCTGCTGATTGGT<br>CTGGCGAAGCGTTTCAAAGAGAGCCCGTTTGAGCTGGAAGATTTCATC<br>CCGATGGACAGCACCGTGAAGAACTATTTTATTACCGATGCGCAGACC<br>GGCAGCAGCAAATGCGTGTGCAGCGTTATCGACCTGCTGCTGGACGA<br>TTTCGTTGAAATCATTAAAAGCCAAGATCTGAGCGTGGTTAGCAAGGTG<br>GTTAAAGTGACCATCGATTACACCGAGATTAGCTTTATGCTGTGGTGCA<br>AGGACGGTCACGTGGAAACCTTCTATCCGAAACTGCAATAA

Supplementary References

1 Scheres, S. H. & Chen, S. Prevention of overfitting in cryo-EM structure determination. *Nat Methods* **9**, 853-854, doi:10.1038/nmeth.2115 (2012).

2 Rosenthal, P. B. & Henderson, R. Optimal determination of particle orientation, absolute hand, and contrast loss in single-particle electron cryomicroscopy. *J Mol Biol* **333**, 721-745 (2003).

3 Punjani, A., Rubinstein, J. L., Fleet, D. J. & Brubaker, M. A. cryoSPARC: algorithms for rapid unsupervised cryo-EM structure determination. *Nat Methods* **14**, 290-296, doi:10.1038/nmeth.4169 (2017).

4 Pei, J., Kim, B. H. & Grishin, N. V. PROMALS3D: a tool for multiple protein sequence and structure alignments. *Nucleic Acids Res* **36**, 2295-2300, doi:10.1093/nar/gkn072 (2008).

5 Waterhouse, A. M., Procter, J. B., Martin, D. M., Clamp, M. & Barton, G. J. Jalview Version 2--a multiple sequence alignment editor and analysis workbench. *Bioinformatics* **25**, 1189-1191, doi:10.1093/bioinformatics/btp033 (2009).

6 McLuckey, S. A., Van Berkel, G. J. & Glish, G. L. Tandem mass spectrometry of small, multiply charged oligonucleotides. *J Am Soc Mass Spectrom* **3**, 60-70, doi:10.1016/1044-0305(92)85019-G (1992).
