# Supplementary material for: Cryo-EM structures of the SARS-CoV-2 endoribonuclease Nsp15 reveal insight into nuclease specificity and dynamics
Source: Nat Commun. 2021 Jan 27;12:636. doi: 10.1038/s41467-020-20608-z (PMC7840905; doi:10.1038/s41467-020-20608-z)
Supplement: Supplementary file 3 — Description of Additional Supplementary Files [file 41467_2020_20608_MOESM3_ESM.pdf]

# Description of Additional Supplementary Files

**Supplementary Movie 1**. Cryo-EM reconstruction of nucleotide bound Nsp15

**Supplementary Movie 2**. 3D Variability of Apo-H235A-Nsp15 dataset i

**Supplementary Movie 3**. 3D Variability of Apo-wt-Nsp15

**Supplementary Movie 4**. 3D Variability of Apo-H235A-Nsp15 dataset ii
