# Supplementary material for: Cryo-EM structures of the SARS-CoV-2 endoribonuclease Nsp15 reveal insight into nuclease specificity and dynamics
Source: Nat Commun. 2021 Jan 27;12:636. doi: 10.1038/s41467-020-20608-z (PMC7840905; doi:10.1038/s41467-020-20608-z)
Supplement: Supplementary file 8 — Reporting Summary [file 41467_2020_20608_MOESM8_ESM.pdf]

nature research

Corresponding author(s): Robin E. Stanley and Monica C. Pillon

Reporting Summary

Nature Research wishes to improve the reproducibility of the work that we publish. This form provides structure for consistency and transparency in reporting. For further information on Nature Research policies, see Authors & Referees and the Editorial Policy Checklist.

Statistical parameters

When statistical analyses are reported, confirm that the following items are present in the relevant location (e.g. figure legend, table legend, main text, or Methods section).

n/a	Confirmed	
☐	☒	The exact sample size (*n*) for each experimental group/condition, given as a discrete number and unit of measurement
☐	☒	An indication of whether measurements were taken from distinct samples or whether the same sample was measured repeatedly
☐	☒	The statistical test(s) used AND whether they are one- or two-sided<br>*Only common tests should be described solely by name; describe more complex techniques in the Methods section.*
☒	☐	A description of all covariates tested
☒	☐	A description of any assumptions or corrections, such as tests of normality and adjustment for multiple comparisons
☐	☒	A full description of the statistics including central tendency (e.g. means) or other basic estimates (e.g. regression coefficient) AND variation (e.g. standard deviation) or associated estimates of uncertainty (e.g. confidence intervals)
☐	☒	For null hypothesis testing, the test statistic (e.g. *F*, *t*, *r*) with confidence intervals, effect sizes, degrees of freedom and *P* value noted<br>*Give P values as exact values whenever suitable.*
☒	☐	For Bayesian analysis, information on the choice of priors and Markov chain Monte Carlo settings
☒	☐	For hierarchical and complex designs, identification of the appropriate level for tests and full reporting of outcomes
☒	☐	Estimates of effect sizes (e.g. Cohen's *d*, Pearson's *r*), indicating how they were calculated
☐	☒	Clearly defined error bars<br>*State explicitly what error bars represent (e.g. SD, SE, CI)*

Our web collection on statistics for biologists may be useful.

Software and code

Policy information about availability of computer code

Data collection	Cryo-EM data was collected with Serial EM 3.8 beta.
Data analysis	MotionCorr 2, Cyro Sparc V2, and CTFFIND 4 were used for cryo-EM image processing and structure determination.<br>Phenix 1.16-1.18, CCP4MG 2.2.10, Coot 0.8-0.9 and MolProbity 4.5 were used for model building, refinement and validation.<br>UCSF Chimera 1.14, UCSF Chimera X 1.0, and Pymol V2.2.2 were used for visual presentation of the Cryo-EM maps and models.<br>AMBER.18 was used for Molecular Dynamics.<br>Excel V16 was used for statistical analysis.<br>Jalview 2.11 and Clustal Omega were used for protein sequence alignment and visualization.

For manuscripts utilizing custom algorithms or software that are central to the research but not yet described in published literature, software must be made available to editors/reviewers upon request. We strongly encourage code deposition in a community repository (e.g. GitHub). See the Nature Research guidelines for submitting code & software for further information.

Data

Policy information about availability of data

All manuscripts must include a data availability statement. This statement should provide the following information, where applicable:

- Accession codes, unique identifiers, or web links for publicly available datasets
- A list of figures that have associated raw data
- A description of any restrictions on data availability

The EM maps have been deposited in the EMDB under the following accession codes EMD-22610, EMD-22611, EMD-22612 and EMD-22613. The atomic coordinates for Nsp15 bound to UMP have been deposited to the PDB under the following accession code PDBID-7K0R. Raw data from the nuclease assays in Figure 2d are provided as a supplementary source data file. All other data and constructs used in this study will be made available upon request addressed to R.E.S. (robin.stanley@nih.gov) and M.C.P (monica.pillon@nih.gov).

Field-specific reporting

Please select the best fit for your research. If you are not sure, read the appropriate sections before making your selection.

☒ Life sciences ☐ Behavioural & social sciences ☐ Ecological, evolutionary & environmental sciences

For a reference copy of the document with all sections, see nature.com/authors/policies/ReportingSummary-flat.pdf

Life sciences study design

All studies must disclose on these points even when the disclosure is negative.

Sample size	Each 3D cryo-EM reconstruction was calculated from 100s of micrographs containing Nsp15 particles. The number of particles varies per dataset as it is dependent upon the density of particles per micrograph and the number of micrographs collected.
Data exclusions	During cryo-EM data processing poor micrographs and bad particles were discarded following 2D and 3D classification.
Replication	Each cryo-EM map was created from one data set. We determined three cryo-EM reconstructions of apo Nsp15, which all yielded similar 3D maps with variability in the endoU domain. All RNA cleavage assays were performed with at least three technical replications from multiple independent protein purifications. We also performed three independent replicates of the MD simulations.
Randomization	Nsp15 particles were randomly selected using CyroSparc for an initial round of 2D classification. Particle picking was then subsequently subject to a template-based approach.
Blinding	Blinding was not relevant to this study as there are no experiments subject to bias.

Reporting for specific materials, systems and methods

Materials & experimental systems

n/a	Involved in the study
☐	☒ Unique biological materials
☒	☐ Antibodies
☒	☐ Eukaryotic cell lines
☒	☐ Palaeontology
☒	☐ Animals and other organisms
☒	☐ Human research participants

Methods

n/a	Involved in the study
☒	☐ ChIP-seq
☒	☐ Flow cytometry
☒	☐ MRI-based neuroimaging

Unique biological materials

Policy information about availability of materials

Obtaining unique materials	All data and constructs used in this study will be made available upon request.
